# State Medicine*Abstracts from an address to the graduating class of the Medical Department of the University of Buffalo, Feb. 25, 1880.

**Published:** 1880-04

**Authors:** Thomas F. Rochester

**Affiliations:** Professor Principles and Practice of Medicine


THE BUFFALO

MEDICAL AND SURGICAL JOURNAL.

VOL. XIX.—APRIL, 1880.—No. 9.

### ORIGINAL COMMUNICATIONS.

### STATE MEDICINE.*

BY THOMAS F. ROCHESTER, M. D., PROFESSOR PRINCIPLES AND PRACTICE OF MEDICINE.

ONCE every year there are gathered together in this hall a pleased and ambitious class, who, having passed the requisite examination, receive from the proper authority the degree of Doctor of Medicine. This is attested by a legalized document, called a diploma, which certifies that the individual whose name it bears is authorized to practice medicine, and to perform surgical operations. Is this its only significance? It is hoped not, as the speaker will endeavor to show, not only to the class, but also to the audience, whom he addresses over their heads. He has selected as his topic, State Medicine, a theme which is as important and as intelligible to the one as to the other. It is not clothed or hemmed in by any technicalities, and is readily comprehended by every person of reasonable information. It is by no means confined to the domain of medicine, it is intimately affiliated with law and depends greatly upon the moral

*Abstracts from an address to the graduating class of the Medical Department of the University of Buffalo, Feb. 25, 1880.

teaching and support of religion. The learned professions all walk within its circle, and never collide. It is not, however, limited to these professions. It embraces much that pertains to science, art, invention, labor and to national, state and municipal authority. It may be broadly defined, as that science which aims to prevent, remove or mitigate the action of all agents tending to impair health, shorten life, affect the intellect or degrade the moral sense. It is in truth mental or physical sanitation, the former being greatly dependent on the latter, as evinced by the numberless agencies which dethrone the reason and which is restored to its balance and power, chiefly by hygienic appliances, and by the removal of the individual from his customary surroundings. Never was there a truer maxim than the trite one of "*Mens sana in corpore sano.*" The old idea that mind was something independent of matter, is no longer tenable, and the psychologist of the present day is not a theorist or rhapsodist, as were his predecessors, but of necessity a physiologist and pathologist, who reasons from material principles and facts, and makes his deductions from established bases. This illustration of cause and effect is here introduced to indicate what kind of argument is to be presented for your consideration, the more so as mania was formerly regarded as occult and mysterious, and even a wrathful visitation of Divine Providence, now happily rescued by science from any such unnatural and unjust imputation. It is, moreover, one of the proudest trophies of the domain of state medicine, for as yet, in nothing has it been of such signal service as in improving the treatment of the insane. No school of medicine is complete that has not its psychological department, and such it is hoped will be founded this year in the University of Buffalo, now that our city contains an ample and most perfect asylum.

It is the boast of the present century that human life is growing longer, and this is attributed to the effects of an advancing knowledge of many morbific influences and the consequent avoidance of the same as far as is possible and practicable.

And yet, while this is true, there are perils developed and fostered by the very agencies which are intended to be sanitary, and which are so when properly managed and directed. As an illustration, take the matter of drainage. Not so very long ago many diseases were claimed to be intensified and even originated by certain agents upon and within the ground, and the remedy indicated and demanded was drainage. Sewers were constructed and now the same disorders are ascribed to sewer gas. Admitting that this may be in part true, it does not disprove the former assertion. It only shows that the process has not gone far enough. It remains to destroy or remove the pestilential gases by action upon them within the sewers, and not only this, but the solid and fluid contents of the sewers should be discharged into reservoirs, and there disinfected before being allowed to pass into any stream or water-course. This is now being done most successfully in England, and the solid residuum, after being rendered innocuous, commands a high price for enriching soil employed for agricultural purposes, the disinfecting and purifying agents being a mixture of sulphate of iron and sulphate of alum, the cost of which is so trifling that it adds but very slightly to the expense of the mechanical apparatus and manual labor required. With our municipal authorities this should especially be borne in mind at the present time, when after much discussion and opposition the much needed Bird Avenue sewer is in process of construction. Not only should all public, but all private drains be well ventilated, and the gases carried far above all usual habitable elevation. This is easily done in private houses by having the water-conductor empty into the sewer, creating during rains a powerful wash-out, and at other times a passage for gases at least as high as the eaves. Besides this every trap should be ventilated by pipe communication with a chimney flue ; for a water trap, be it ever so tight and well constructed, does not absorb and hold the noxious emanations of the drain. When this cannot be done, permanent washstands, closets and bath tubs should be dispensed with. Many an insidious disease and low fever can be traced to the so-called modern improvements. The water trap may deodorize but not destroy, and thus render the more dangerous that which the sense of smell cannot detect and therefore guard against. A good thing should not be condemned, however, because it has imperfections; these will always be detected and corrected. Ancient Rome had abundant public baths and magnificent sewers, the latter still extant, and they elicit the wonder and admiration of the modern beholder. Yet Rome was, and is, one of the unhealthiest of cities; but, on the other hand, look at Paris and London, especially the latter, those great hives of human beings, formerly very pest holes, now perhaps the most salubrious cities on earth, and made so by their drainage. London was a bed of malaria, and hundreds of its citizens yearly succumbed to its deadly effects, among others the great protector, Oliver Cromwell. Rome was made only less unhealthy, but Paris and London were sanitarily revolutionized.

In this connection it seems appropriate to consider briefly water supply. It has been demonstrated often and in many places, all over the inhabitable globe, within the last quarter of a century, science being the investigator, that the clear and sparkling spring, and that the deep well with its fountain of cold, bright and inodorless water, often contains and disseminates poison more deadly and rapid than those sown and distributed by the demon of alcohol; and this especially within the cofines of cities and villages and more rarely on isolated farms. Let not our good temperance friends stand aghast at this declaration. Water is intrinsically healthful; alcohol intrinsically the reverse; but the former, under certain circumstances, becomes contaminated by disease germs which it distributes. The speaker here refered to Dr. Flint's observations thirty-five years ago. Since that time many observers have given to the medical world indisputable evidence of typhoid water contamination and distribution, and lately none more notably and positively than Prof. E. S. Van de Warker of Syracuse, who shows in the *Popular*

*Science Monthly* of 1879, how through one person seventeen others became ill from a well poisoned by want of proper drainage in its vicinity. But it is proper to state here that typhoid fever will never emanate from water supply, however foul and impure, unless the special typhoid germ is conveyed to it, and also to state that no water, however pure, may not be made a vehicle for it; and this is adduced as an argument, if such is needed, for thorough and efficient drainage, which may be made so complete as to do away with soil saturation. The wells of this city were many of them examined within two years by a competent chemist. The water of very few of them was suitable for drinking purposes. They were subject to soil saturation; this does not mean that drains or vaults emptied into them directly or indirectly, although even such accidents have happened; it means the reception of improper material by percolation through the soil. This was shown by the excess of chlorine and of albumenoid ammonia, in many instances being considerably more than that found in sewer water itself; and yet to a casual observer this water was clear, cold, tasteless and inodorless. That these wells originate many diseases and aggravate more there can be no possible doubt. Prof. Breneman of Cornell University, on this subject says: "It shows the extent of soil saturation and indicates the condition of the ground, the emanations and exhalations from which taint the air in which the families are obliged to live, and expose them to a constant morbific agency." For this the only remedy is drainage; and the drain? Ventilate it, wash it out often, and when it discharges its contents, disinfect them and make some portion useful, and carry off the remainder, now made harmless by artificial or natural channels. Cold and deep and rapid as is our noble Niagara, it should not be tinctured with any uncleansed scourings. At one time, but a few years back, our reservoir received a percentage of the outpouring of the canal and harbor. Various intestinal troubles were very prevalent. Now the tunnel has been carried far out into the stream, and these disorders are relatively rare.

At one of our anniversaries, some time ago, the speaker dwelt at some length upon the heating, lighting and ventilation of dwellings, churches and public schools, it is believed, with good effect. Similar appeals made here and elsewhere by various persons, called public attention to many unsuspected evils, which are now either removed or are in process of correction. It is not proposed to go over this ground again, except to say that in the residence, sanitation in the cellar and bedroom are of paramount importance.

Certain mortuary practices exist which demand strict inquiry and limitation. Reference is made to ice surrounding and so-called embalming of bodies. These are both to be commended under proper restrictions and limitations, but without them, as now practiced, are utterly wrong, may be life-destructive, and in conflict with necessary judicial inquiry. They should never be used except with the knowledge and consent of the attending physician; yet how rarely is this the case. No one can decide upon the reality of death, or of its probable cause, but an intelligent and educated medical man; but often his opinion is not asked. The patient whom he last saw living is enclosed in ice at his next visit. Who knows that the frozen and lifeless body was not in a state of syncope or trance. There is no excuse and no necessity for this indecent haste, and the undertaker who counsels and practices it, may be the cause of the death which he presumptuously assumes has taken place. Embalming, which consists of injecting arsenic and other antiseptic agents into the system, still less should be practiced, without competent medical authorization. It interferes most seriously with all subsequent pathological research, and may cover up crime and prevent the detection and punishment of its perpetrators. No chemist can determine the question of poisoning when this operation has been made. Had Hotchkiss been "embalmed" Lewiston and Lockport would have escaped their present excitement. It is said that the New York Medico-Legal Society has taken up this matter and is initiating steps to regulate this process by much needed legal enactment.

All of, you remember the fearful visitation of the south and southwest, by yellow fever, two years ago. It caused the appointment of the National Board of Health, and last year the wisdom of the creation of such a body was demonstrated by the limitation of the pestilence to sadly unfortunate Memphis. Through its advice the most rigid quarantine was enforced in and about the disease-infested and beleagured city, and the result was that the plague was fenced in, and yet the year before, when quarantine was advocated at the meeting of the American Medical Association, at Atlanta, the proposition was most violently opposed by the medical men present from New Orleans. Last summer's experience ought to, and probably will, silence such hypothetical and factious resistance in the future. This Board has now in contemplation another step striking at the very root of the matter. It proposes to destroy the disease at its birth-place by coöperating with the Cuban authorities in cleansing and disinfecting the harbors of Havana and contiguous ports. The experiment is one of great magnitude, and will be watched with absorbing interest. If successful, it will save yearly thousands of lives and promote commerce enormously, and will cause similar procedures to be adopted in many West Indian and South American seaboard cities. Cholera, which has heretofore been considered a mysterious emanation from the jungles of Burmah and Siam, is now thought to be due to the impure water employed for drinking; and the British Government, always foremost in sanitary reforms, has appointed a commission to investigate the grounds for this assertion which, if found to be true, will open the way for avoiding one of the most terrible scourges of the human race. But while attention is given to these fell giants of destruction, all communities should be taught that many disease-fostering and developing agencies are entirely under individual and corporate and municipal control. Slaughter-houses should never be allowed to contaminate the air and the soil of city or village. How completely this can be prevented; witness the immense cleanly and inodorous abbatoirs of Paris, where no blood or intestinal products become scources of pollution, where everything is either destroyed or made useful in art or agriculture. Still less should large numbers of animals be congregated in confined quarters for sale or for feeding and fattening, as is done in this and other cities. Within the year an enormous pigstye attached to a grape sugar factory, in a thickly populated quarter of Buffalo, offends the nostrils and jars upon the ears of all in the vicinity. Such nuisances should not be tolerated in any civilized community. If not positively unhealthy, they are certainly very objectionable, and the fat, soggy pork produced by such stuffing is presumably not of the most nutritious or palatable quality. It is well known that a certain proportion of hogs are the subjects of a parasitic microscopic disease caused by the trichina spiralis, which is produced and reproduced in great numbers and is found in all parts of their bodies. It is very tenacious of life and is not destroyed by smoking or pickling, and when cooked, only by long continued and extreme heat. The rat is the habitat of the same parasite, and it has been suggested that it is conveyed to the omnivorous swine by eating these pests. The trichina has been found in clean, isolated and well fed hogs, but it is probable that the larger number infected are from the animals fattened in connection with distilleries and grain product factories, where the rat is always in great numbers. The hog does not appear to suffer from the parasite, but it produces in the human being who eats of his infected flesh, blood poisoning and low fever, attended with very large mortality. The deduction from this is very clear. No fresh pork should, under severe penalty, be passed into the hands of dealers without an inspection certificate that it is free from trichina. The inspectors should be appointed by proper authorities; any intelligent person with fair eyesight can discharge the duty—a barbed hook and a cheap microscope being the only requisite appliances. But meat inspection should not be limited to pork. We all know by report at least, of the Texas cattle disease and of epidemic pleuro-pneumonia. Animals thus affected, die or are killed and some of their carcasses are destroyed, but it is to be feared that many of them find there way to the stalls. The flesh of healthy cattle that are overdriven, or worn out by long railway travel without suitable supply of water or food, or that are much bruised or trampled upon, does not keep well, and yet a great deal of this is sold, who can say at what cost of human suffering and sickness. There are in most cattle yards regulations to prevent these evils, and there are city ordinances to the same effect, but rarely are they enforced, and rarely are the penalties for violations inflicted. Under this head comes the consideration of milk supply, especially of cities. The proverbial pump dilution is not altogether a question of dollars, if what has already been said about city wells is borne in mind.

In examining the last annual reports of the various city and State Boards of Health, we are struck with the marked increase in the variety and value of their contents. The contributions cover an unusally wide ground and show that sanitary science can be brought to bear not only upon social and economical, but even upon literary and artistic matters. At the present rate, indeed, hygienic law and studies will soon interpenetrate every phase and period of a civilized man's existence. The State sanitarians have already struck at the very beginnings of life. They have introduced themselves to the infant and have mingled with scientific data the uncertain joys that attend his growth. The last Massachusetts Report proposes that the puerperal woman be at once supplied with printed forms upon which to record the physical condition and progress of her offspring. Such a plan, it is stated, has been in existence for some time in certain European cities. As the future citizen develops, his course is attended with a constantly increased watchfulness, a watchfulness that does not forget the collection of the more abstruse scientific facts. Nearly every State Board has examined the school houses, and has recommended, or caused to be adopted, properly ventilated rooms. The school-boy is given 250 cubic feet of air where he used to have 25. His eyes are examined, his shoulders kept back and the light thrown in from behind. In Boston the boys and girls have been subjected to anthropometrical investigation, and charts are given, showing how fast the children grow under the high intellectual tension of that city. In the report of the Wisconsin Board is a valuable paper upon the proper reading for the young; nor upon perusal can we do else than believe that State advice and even State regulation is greatly needed in this direction. The class of literature which includes such works as the Half-Dime Novels, works which have the odor and suggestion of an intellectual emesis effects in time quite as much physical evil as imperfect sewers. It is evidence of breadth of view and earnest purpose that scientific bodies speak authoritatively for the State on such subjects. It has from the first been a prominent purpose of state medicine to protect children from the contagious diseases to which they are subject. With each year more potent methods of prophylaxis have been devised, and their application has been more widely urged. The recommendation for the prevention of scarlet fever and diphtheria issued by some of the Boards, and we may refer particularly to those of Massachusetts and Wisconsin, have been valuable additions to medical science. They have been widely circulated, and, as is shown by many letters in regard to them, have excited much interest among the people. This has in turn reflected upon the State Boards; it has made them more popular, and the local town and city Boards more numerous. In Massachusetts sixteen of the nineteen cities, and three hundred and one of the three hundred and twenty-five towns, have organized local Boards of Health, and these make regular reports to the Central or State Boards.

There is, however, no single direction in which Sanitary Boards have made more important advances than in those of vital statistics. The collection of such information is a comparatively recent undertaking in this country, and, as would naturally be the case, has been attended with much difficulty. A busy and practical people cannot easily be made to see the importance of registering every birth, marriage and death. Nevertheless, a foothold has been gained, the sentiment is becoming favorable to such registrations, and already even in some of our Western States very full reports come in each year. We have learned even thus soon, through this work, something of the way in which our nation is growing. Some of the facts have an especial interest to medical men. It is shown that the birth rate among our native born population in the cities is approximately only 16.74 per thousand, while that of the foreign born citizens is 35.23 per thousand. This birth rate for native Americans is even smaller than that of the French in France. Physicians know best, perhaps, the true " inwardness " of this state of things; and as regards remedy, although clergymen can preach and statesmen legislate, it is the medical profession, that can be of the more direct use in educating people to the beauties of a larger domestic circle, or, at least, in discouraging the means for limiting it.* Can this be true, oh! men and women of America? Is the seed of the foreign born to crowd your scanty progeny to the wall? Is your manhood failing, and are your motherly attributes perishing? Is the hearthstone to become cold, and are the attractions and attachments, and the moral influences of a large home circle to be dispensed with? If so, oh! pity for our people and our nation, and infamy and ruin here and hereafter for those base creatures who disgrace and degrade and dishonor the noble profession of medicine by lending themselves to the furtherance of this terrible iniquity. It is unnecessary to speak more plainly or more at length upon this subject, except to say, if there be one field for reform that is larger and more urgent than others, it is this, this reckless and careless and disgraceful, self-willed, selfish native depopulation and consequent demoralization.

* N. Y. Medical Record.

And now, members of the graduating class, and kind friends here present, who have come to see you enter upon your professional path and to wish you joy and success in your career, is it necessary to demonstrate further that much of your usefulness, if you aim to be really beneficial to the world, lies outside of the employment of drugs and surgical appliances? The very fact that you are physicians, places you in the van and will cause you to be consulted in all matters pertaining to sanitary procedures. It is your duty to see that you are fit for and can hold that position, attributed to you by virtue of your profession. To do this you will find it necessary to enlarge your reading and expand your thoughts. You must investigate to a reasonable extent much that pertains to various arts and sciences, to agriculture, to mechanical and hand labor, to topographical and meteorological conditions and phenomena. In short, you must devote much of your time to what is perhaps erroneously termed extra professional study and investigation, and this will repay you in more ways than you perhaps imagine. In the first place it will give you the personal delight of increased knowledge, it will give you depth and breadth, and expansion of thoughts and of ideas. It will make you searching and yet more charitable in your observations. It will increase your mental and your physical strength, and thus by developing your common sense, will in every way enure to your advantage. A thoroughly educated physician, in its true sense, means a thoroughly educated man, and therefore, those of you who aspire to win distinguished and deserved professional renown, will find that with this, the termination of your pupilage, your student's life has just commenced, and it will be co-existent with your period of usefulness on earth. Let your motto be, honor and honesty, sobriety and diligence, and you will not, you cannot, fail.

